# Activated Human Nasal Epithelial Cells Modulate Specific Antibody Response against Bacterial or Viral Antigens

**DOI:** 10.1371/journal.pone.0055472

**Published:** 2013-02-06

**Authors:** Chiou-Yueh Yeh, Te-Huei Yeh, Chiau-Jing Jung, Pei-Lin Chen, Huei-Ting Lien, Jean-San Chia

**Affiliations:** 1 Graduate Institute of Immunology, College of Medicine, National Taiwan University, Taipei, Taiwan; 2 Department of Otolaryngology, National Taiwan University Hospital, College of Medicine, National Taiwan University, Taipei, Taiwan; 3 Graduate Institute of Microbiology, College of Medicine, National Taiwan University, Taipei, Taiwan; The Ohio State University, United States of America

## Abstract

Nasal mucosa is an immune responsive organ evidenced by eliciting both specific local secretory IgA and systemic IgG antibody responses with intra-nasal administration of antigens. Nevertheless, the role of nasal epithelial cells in modulating such responses is unclear. Human nasal epithelial cells (hNECs) obtained from sinus mucosa of patients with chronic rhinosinusitis were cultured in vitro and firstly were stimulated by *Lactococcus lactis* bacterium-like particles (BLPs) in order to examine their role on antibody production. Secondly, both antigens of immunodominant protein IDG60 from oral *Streptococcus mutans* and hemagglutinin (HA) from influenza virus were tested to evaluate the specific antibody response. Stimulated hNECs by BLPs exhibited a significant increase in the production of interleukin-6 (IL-6), and thymic stromal lymphopoietin (TSLP). Conditioned medium of stimulated hNECs has effects on enhancing the proliferation of CD4+ T cells together with interferon-γ and IL-5 production, increasing the costimulatory molecules on dendritic cells and augmenting the production of IDG60 specific IgA, HA specific IgG, IgA by human peripheral blood lymphocytes. Such production of antigen specific IgG and IgA is significantly counteracted in the presence of IL-6 and TSLP neutralizing antibodies. In conclusion, properly stimulated hNECs may impart immuno-modulatory effects on the antigen-specific antibody response at least through the production of IL-6 and TSLP.

## Introduction

Nasal epithelial cells (hNECs) located at mucosal surface serve as the first barrier to microbial challenge and are permissive to drug or vaccine delivery [1], [2]. Epithelial cells are capable of producing various cytokines, chemokines, and growth factors by recognizing microbial-associated molecular patterns (MAMPs) from colonizing microbes or invading pathogens through pathogen recognition receptors, such as Toll-like receptors (TLRs). These factors can induce a local inflammatory response that is characterized by the recruitment and activation of dendritic cells (DCs) [3]. For example, chemokine (C-C motif) ligand 20 (CCL20) can recruit DCs as well as T and B lymphocytes [4], [5], while thymic stromal lymphopoietin (TSLP) can directly activate DCs by upregulating co-stimulatory molecules such as CD40, CD80, and CD86 to promote Th2 cell differentiation [6]. Furthermore, stimulated epithelial cells can produce B-cell-activating factor of the TNF family (BAFF)/B lymphocyte stimulator (BLys), and a proliferation inducing ligand (APRIL) to promote the activation, differentiation, and survival of B cells [7]. Therefore, mucosal epithelial cells may efficiently detect and respond to external antigenic stimulation and bridge with the protective adaptive immune response. Such interactions also underlie the fundamental basis for using mucosal adjuvants to enhance antibody production, which is similar to intestinal epithelial cells interacting with bacterial toxins (e.g. Cholera toxin or *Escherichia coli* enterotoxin) [8], [9] or peptidoglycan derivate muramyl dipeptide (MDP) [10]. However, the capacity of human nasal epithelial cells to mediate or modulate inflammatory reactions in the context of antibody generation is unclear [3].

We have established a system for culturing human primary nasal epithelial cells *in vitro* to subsequently harvest well-differentiated hNECs, as determined by cililary differentiation [11], which express both TLR2 and TLR4 [2]. We previously demonstrated that immunodominant glycoprotein 60 (IDG60) from oral commensal *Streptococcus mutans* is an immunodominat antigen that elucidates a relatively high secretory IgA, serum IgG, and memory CD4+ T cell proliferative responses in the general population [12], [13]. Interestingly, this bacterial protein antigen can noncovalently bind to the bacterium-like particles (BLPs) derived from *Lactococcus lactis* [14], which consist of bacterial peptidoglycan spheres that lack other intact cell wall components and intracellular materials. BLPs are considered to be good carriers for delivering antigens for mucosal immunizations [15], [16], since BLPs induce co-stimulatory molecule expression and proinflammatory mediator production in human CD11c+ DCs through TLR2 activation [17]. In this study, we investigated the *in vitro* immuno-modulatory effect of BLPs-stimulated primary cultured hNECs on the specific antibody production using both *S. mutans* IDG60 and influenza virus hemagglutinin (HA) as tested antigens. The immuno-modulatory effect of BLPs-stimulated nasal epithelium on the IDG60-specific antibody response was also examined in a mouse model.

## Materials and Methods

### Ethics Statement

The isolation and culture of the human nasal epithelial cells used in this study was approved by the ethical committee at the National Taiwan University Hospital. Each patient provided informed written consent.

### BLP and Antigens

BLPs from fresh cultures of *L. lactis* MG1363 cells [18] (kindly provided by Kees Leenhouts, Mucosis BV, 9713 GX Groningen, The Netherlands) were prepared and characterized as previously described [19]. The recombinant IDG60 with His-tag (rIDG60) was expressed in *S. mutans* and purified as previously described [13]. Binding of rIDG60 to BLPs was determined by SDS-PAGE followed by western blot analysis. Influenza virus hemagglutinin (HA, subtype H1; kindly provided by Dr. Li-Min Huang, Division of Infectious Diseases, Department of Pediatrics, National Taiwan University Hospital) was also previously described [20].

### Human Nasal Epithelial Cell and Intestinal Cell Line Cultures

Nasal sinus mucosa was obtained from patients undergoing endoscopic sinus surgery. A statement of informed consent was obtained from each patient and approval for use of human specimens was granted by the National Taiwan University Hospital Committee for Regulation of Human Specimens and Volunteers. The isolation and culture conditions of hNECs were previously described [11]. Briefly, mucosa tissue was treated with 2 mg/ml pronase overnight, pre-plated onto plastic dishes to eliminate most of the contaminating fibroblasts, and enriched in DMEM-F12/Bronchial epithelial cell growth medium (Cambrex Bio Science Walkersville, Inc.). The well-differentiated hNECs (ciliated with transepithelial electroresistance) were used for further activation experiments [11].

HT-29 cells, a human colonic epithelial cell line from ATCC (HTB-38) and CaCO2, a human ileocaecal epithelial cell line from ATCC (HTB-37), were gifts from Dr. Shaw Ning-Sing (Department of Agricultural Chemistry, National Taiwan University) and cultured with DMEM containing 10% fetal calf serum, 2 mM glutamine, and antibiotics.

### Cell Activation and Cytokine Production

Cultured medium with or without BLPs stimulation (1.75×10^8^ BLPs per 3×10^5^ hNECs) was collected to determine IL-6, IL-8, MCP-1, and TSLP production by ELISA (R&D Systems). The expression of these chemokines and cytokines was monitored by real-time PCR using the KAPA SYBR FAST qPCR kit (KAPA Biosystems) as previously described [21]. The specific primers used in this study were indicated in Table S1. Primary culture hNECs from six out of ten batches that exhibited comparable levels of cytokine and chemokine (interleukin 8, monocyte chemoattractant protein-1, and others) production before stimulation were selected for subsequent experiments in this study. Human peripheral mononuclear cells (PBMCs) were prepared by Ficoll-Paque Plus gradient and monocytes were enriched by negative selection (RosetteSep, Stemcell Technologies Inc.). Monocyte-derived dendritic cells (mDCs) were prepared by supplementing the media with GM-CSF and IL-4 (50 ng/ml of each) for six days as previously described [22]. Flow cytometry analysis indicated that the percentage of CD14 and CD1a positive derived immature mDCs was 4.23±0.4% and 96.48±0.64% (mean percentage ± standard deviation), respectively. Activation and maturation of mDC with conditioned medium from BLPs-stimulated hNECs were monitored by expression analysis of CD40 (clone IT2.2) and CD86 (clone 5C3) using a FACSCalibur™ flow cytometer (BD Biosciences). CD4+ T cells were obtained from negative selection with human CD4+ T cell enrichment cocktail (RosetteSep). Proliferation of T cells activated by autologous mDCs plus 1 µg/ml soluble anti-human CD3 (clone OKT3, eBioscience) in the presence or absence of BLP-stimulated condition medium was analyzed by flow cytometry for carboxyfluorescein succinimidyl ester-labeled CD4+ T cells. The supernatant was collected and the IL-2, IL-4, IFN-γ, and IL-5 concentrations were determined. All experiments were conducted in triplicate and results are shown as mean percentages ± standard deviation (SD).

### Antibody Production after in vitro Immunization

The *in vitro* immunization procedure was modified from a previous study [23]. Briefly, isolated PBMCs were treated with 0.25 mM Leu-Leu-OMe (Sigma-Aldrich) for 20 min to remove inhibitory immune cells, such as NK and CD8+ T cells, and were subsequently defined as peripheral blood lymphocytes (PBLs). Human PBLs were sensitized with HA or rIDG60 in the presence or absence of stimulated hNECs conditioned medium or TLR2 ligands, including lipoteichoic acid (LTA, Sigma-Aldrich), peptidoglycan (PGN, Sigma-Aldrich), and BLPs. Blockage experiment was performed with neutralizing antibodies including IL-6 (clone MQ2-13A5, eBioscience), TSLP (clone 15B11.3, BioLegend), and control IgG (clone P3.6.2.8.1, eBioscience) pre-incubated with CM from hNECs stimulated by BLPs for 30 minutes. Immunized PBLs were cultured for seven days and then the supernatant was collected to determine antigen-specific response, Immunoglobulin (Ig) response, and total Ig concentration. Antigen-specific human IgG or IgA was determined by enzyme-linked immunospot assay (ELISpot) with biotin-conjugated goat anti-human IgA α chain or IgG γ chain (Rockland Immunochemicals Inc.), and counted using the CTL ImmunoSpot® Professional Software (Cellular Technology Ltd.). IL-2, IL-4, IL-5, IL-6, IL-8, MCP-1, TSLP, and IFN-γ concentrations in culture supernatants were assayed by sandwich immunoassays using ELISA. Total human IgA and IgG concentrations were determined by the Human IgA and IgG ELISA Quantitation Set (Bethyl Laboratories).

### Nasal Immunization in Mice

Six to 8-week-old female BALB/c mice were purchased from the animal center of National Taiwan University College of Medicine and kept in a specific pathogen-free environment. All animal experiments were approved by the animal care committee of the Medical College of National Taiwan University. Mice were divided in four groups of five mice each. All four groups of animals were immunized intra-nasally on day 0, 7, and 14. Mice were anesthetized and nasally immunized with a 20 µl aliquot (10 µl/nostril) of PBS containing 2 µg of rIDG60 protein alone or BLP-IDG60. IDG60 specific antibodies were measured by ELISA using biotin-conjugated antibodies directed against mouse IgG1, IgG2a, IgG2b, IgG3, or IgE (Bethyl Laboratories, Montgomery, TX). Mice were then euthanized two weeks after the third immunization and the spleen, nasal-associated lymphoid tissue (NALT), and cervical lymph nodes (CLN) were harvested under sterile conditions. A single cell suspension was prepared and cultured with 2 µg IDG60 for 48 hours, and then IL-2 (clone JES6-1A12), IL-10 (clone JES5-16E3), IFN-γ (clone AN-18) and IL-4 (clone 14-7041-85) secreting cells were measured by ELISpot. The results are expressed as the mean number of spot-forming cells per 10^6^ cells.

### Statistical Analysis

The data were analyzed statistically using the unpaired *t*-test with Welch's correction (2-tailed) and two-way ANOVA to compare the mean levels of cytokine secretion or Igs production following a particular treatment. Differences with *p* values <0.05 were considered statistically significant and indicated as *, *p* values <0.01 were indicated as **, and *p* values <0.001 were indicated as ***.

## Results

### Enhancement of Specific Antibody Production by BLPs in a Mouse Model

**Figure 1:**
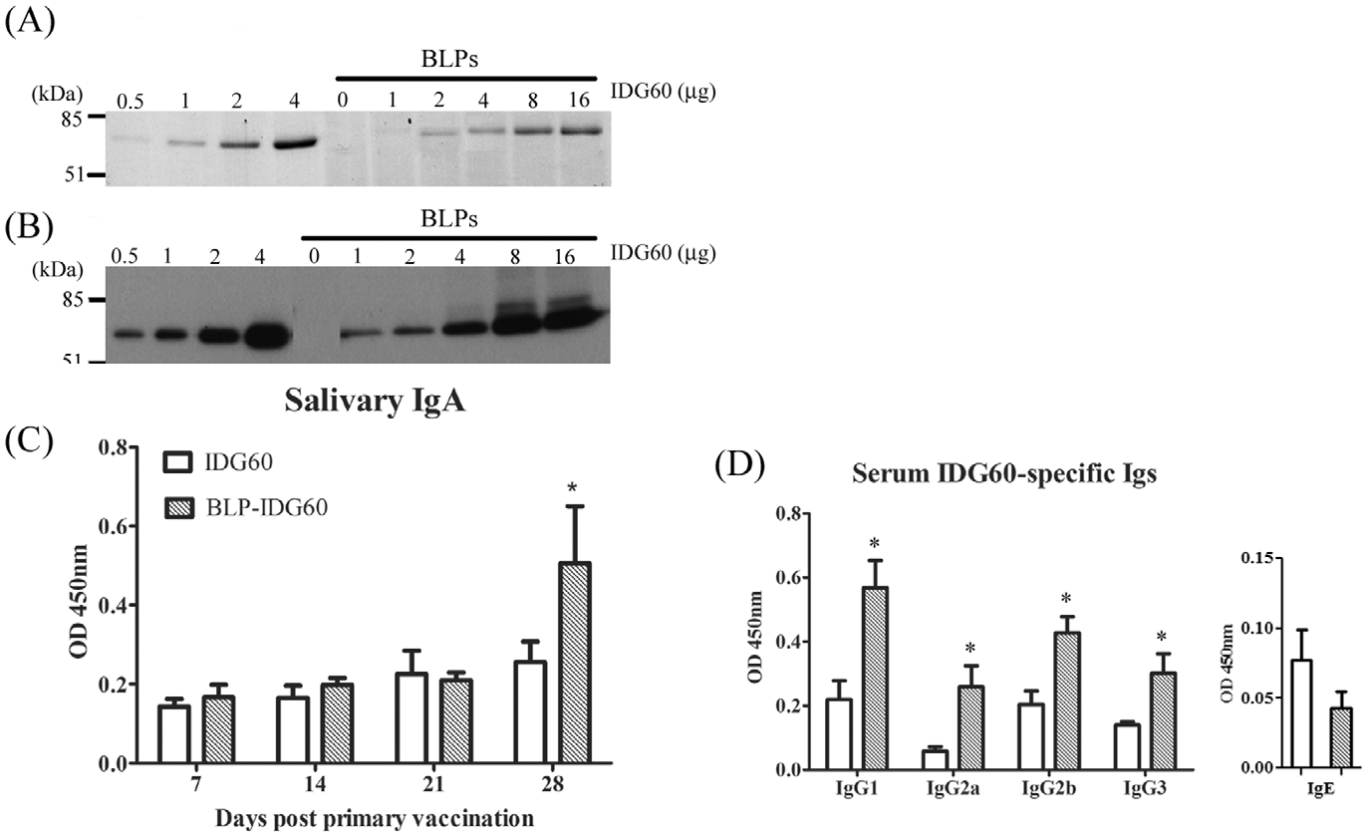
Intranasal immunization of mice with *L. lactis* BLPs elicits potent systemic and mucosal immune responses. Recombinant IDG60 is a peptidoglycan-binding domain bearing protein that was purified from *Streptococcus mutans*. (A) The concentration of IDG60 bound to BLPs was evaluated using SDS-PAGE stained with Coomassie blue and (B) western blot using an anti-His antibody. In the intranasal immunization mouse model, IDG60 protein alone or BLP-IDG60 was administered on day 0, 7 and 14 of the study (arrows). Saliva samples were collected every week and blood was collected on day 28. (C) The kinetics of IDG60-specific salivary IgA levels. (D) The distribution of IgG subclasses and IgE. Significant differences compared with medium-stimulated cells using ANOVA two-way tests are indicated by an asterisk (*p*<0.05).

To confirm the immuno-modulating effect of the BLPs and to test whether such effect on antibody production was dependent on the covalent linkage of the protein antigens with BLPs, IDG60 from oral commensal *S. mutans* was selected as the tested antigen via intra-nasal immunization in a mouse model. Interestingly, protein antigen IDG60 formed high affinity non-covalent bonding onto the surface of BLPs needless further addition of a BLP-specific tag (Fig. 1A and B), which made it possible to investigate in a mouse model the *in vivo* effect of stimulating nasal mucosa *per se* and modulating the systemic antibody production. Saliva samples were taken every week from 7 to 28 days after intra-nasal immunization with soluble IDG60 protein only or BLPs-coupled IDG60 (BLP-IDG60). Higher levels of IDG60-specific IgA responses were detectable only in saliva samples taken 28 days after immunization in mice that received BLP-IDG60 (Fig. 1C). In serum, all IDG60-specific IgG subclasses (IgG1, IgG2a, IgG2b, and IgG3) were elevated in the BLP-IDG60 group; however, IgE did not have an obvious change in either group (Fig. 1D). Furthermore, the IL-2, IL-4, IL-10 and IFN-γ producing cells in nasal-associated lymphoid tissue isolated from the BLP-IDG60 group were elevated by 11.5, 5.0, 9.2 and 1.8-fold, respectively (Table 1). These results indicated that BLPs when intra-nasally administered concomitantly with a protein antigen could enhance the antigen specific antibody response *in vivo*.

**Table 1:** Cytokine secretion profile in spleen, NALT and CLN.

		Spot-forming cells			
		IL-2	IL-4	IL-10	IFN-γ
Spleen^a^	IDG	49.5±2	36.5±3.5	184±9.9	148±59
	BLP-IDG	15±5.6(0.3)	18±1.4(0.5)	71.5±16.3(0.4)	220±7.1(1.5)
NALT^b^	IDG	3.8±1.8	3.2±0.4	2.5±2.85	3±0.7
	BLP-IDG	44±4.2(11.5)^d^	16+4.2(5.0)	23±2.8(9.2)	5.5±0.7(1.8)
CLN^c^	IDG	3.5±0.7	1.5±2.1	3.5±2.1	12±4.2
	BLP-IDG	4.5±2.1(1.3)	2.5±0.7(1.7)	11±2.8(3.1)	12.5±4.9(1.0)

a and^ c^ indicated spots per 10^6^ cells,

b indicated spots per 10^4^ cells,

d indicated the increased fold in BLP-IDG compare to IDG group.

### Stimulated Human Nasal Epithelial Cells Release Inflammatory Mediators in Conditioned Medium

**Figure 2:**
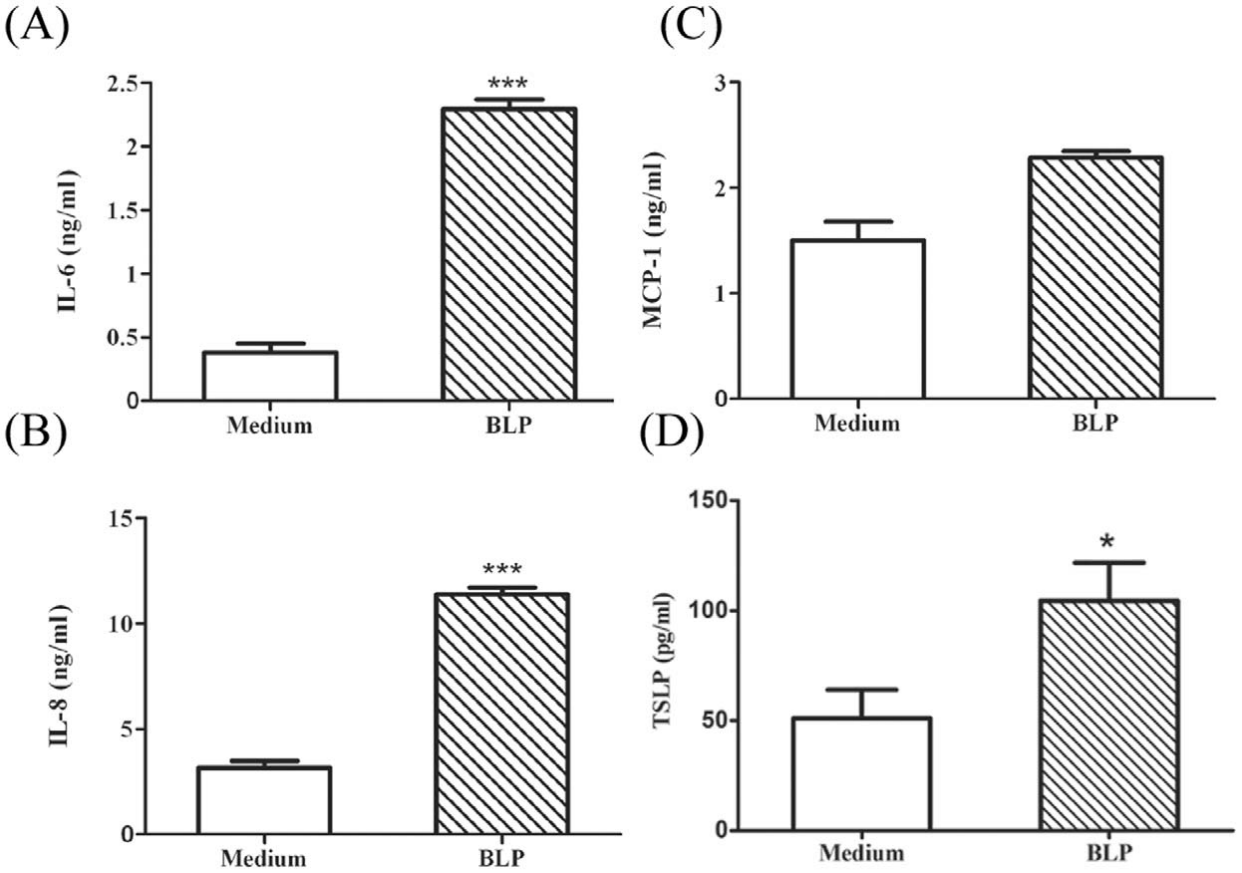
BLP-stimulated hNECs evoke chemokine and cytokine expression. The secretion of (A) IL-6, (B) IL-8, (C) MCP-1 and (D) TSLP by hNECs stimulated with BLPs or medium for 24 h was measured by ELISA. The data are represented as the mean ± standard deviation (SD) from six individuals hNECs performed in triplicate. Significant differences compared with medium-stimulated cells using *t*-tests are indicated by asterisks (*p<0.05 and ***p<0.001).

The immuno-modulating effect of BLPs was subsequently tested on the primarily cultured hNECs exhibiting phenotypically well-differentiated characteristics (ciliated epithelium with transepithelial electroresistance) as previously reported [11]. Conditioned medium and hNECs cell cultures isolated from six different donors were harvested separately after BLPs stimulation for 24 h to determine the production of IL-6, IL-8, or MCP-1 as well as TSLP. There was a significant enhancement in the production of IL-6 and IL-8, with a 6.4- (*p* = 0.0003) and 3.7-fold (*p* = 0.0004) increase, respectively, in the BLPs-stimulated hNECs compared to unstimulated cells (Fig. 2A and B). MCP-1 production was only slightly increased (1.6 fold; *p* = 0.0541; Fig. 2C). The secretion of TSLP in hNECs was up-regulated after BLP stimulation by 2-fold (*p* = 0.0305) compared to unstimulated hNECs (Fig. 2D). The transcription levels of *ccl20* and *tslp* were upregulated 4-fold (*p* = 0.0326) and 5.3-fold (*p* = 0.0423), respectively, by BLPs. In addition, the expression of *tlr2*, which encodes the TLR2 receptor for BLPs in DCs, was slightly upregulated by 2.1-fold (*p* = 0.11) in BLPs-stimulated hNECs (Fig. S1A). The expression of TLR2 on the surface of hNECs was also slightly induced by BLP stimulation (Fig. S1B).

### Conditioned Medium from BLPs-stimulated hNECs Promotes Immune Cell Activation

**Figure 3:**
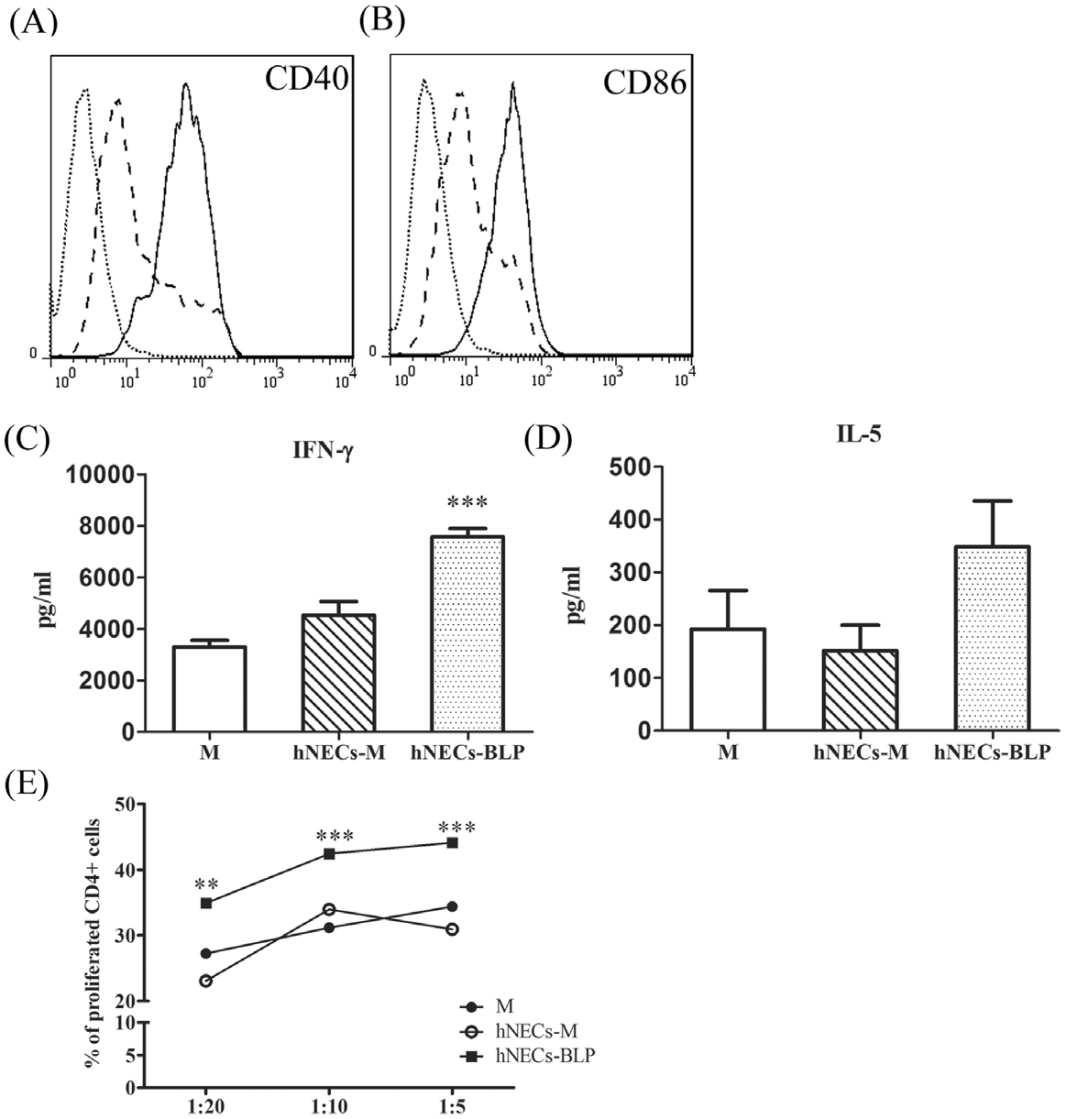
BLPs enhance the maturation of human DCs and the stimulation of T cell proliferation. Expression of (A) CD40 and (B) CD86 on monocyte-derived DCs (mDCs). Human mDCs were stimulated with conditioned medium (CM) from hNECs stimulated with BLPs or medium for 24 h. The data are represented as the percentage of CD40- and CD86-positive cells among total mDCs. The data was shown as isotype control (gray line), medium (dotted line), or conditioned medium of hNECs with BLP stimulation (solid line). (C) Autologous CD4+ T cell proliferation in the presence of human mDCs primed with medium or CM from hNECs stimulated with or without BLPs. (C) IFN-γ and (D) IL-5 were determined by ELISA and represented as mean ± standard deviation (SD). (E) CD4+ T cells proliferation were determined by the carboxyfluorescein succinimidyl ester profiles and were analyzed at 132 h. The data are represented as the percentage of proliferating CD4+ T cells at a DC: T ratio of 1∶20, 1∶10, and 1∶5 from three individuals performed in triplicate. Significant differences compared with medium-stimulated cells using ANOVA two-way tests are indicated with asterisks (**p<0.01 and ***p<0.001).

Conditioned medium from BLPs-stimulated hNECs significantly enhanced the expression of DC maturation markers; co-stimulatory CD40 and CD86 molecules on the surface of human mDCs (Fig. 3A and B). When the activated mDCs were co-cultured with autologous CD4+ T cells plus anti-CD3 and anti-CD28, there was a 2.4- and 1.8-fold increase in the secretion of IFN-γ and IL-5 compared to 1.4- and 0.8-fold in cells cultured with unstimulated mDCs (*p*<0.0001 and *p* = 0.1013, respectively; Fig. 3C and D). The mDCs primed with conditioned medium from stimulated hNECs, but not plain medium, consistently and significantly enhanced proliferation of autologous CD4+ T cells in a ratio-dependent manner (Fig. 3E). In contrast, BLPs alone did not induce the proliferation of either T or B cells, excluding the possible mitogenic effect attributed to BLPs (data not shown).

### BLPs-stimulated hNECs Enhance in vitro Antigen-specific IgA and IgG Production

**Figure 4:**
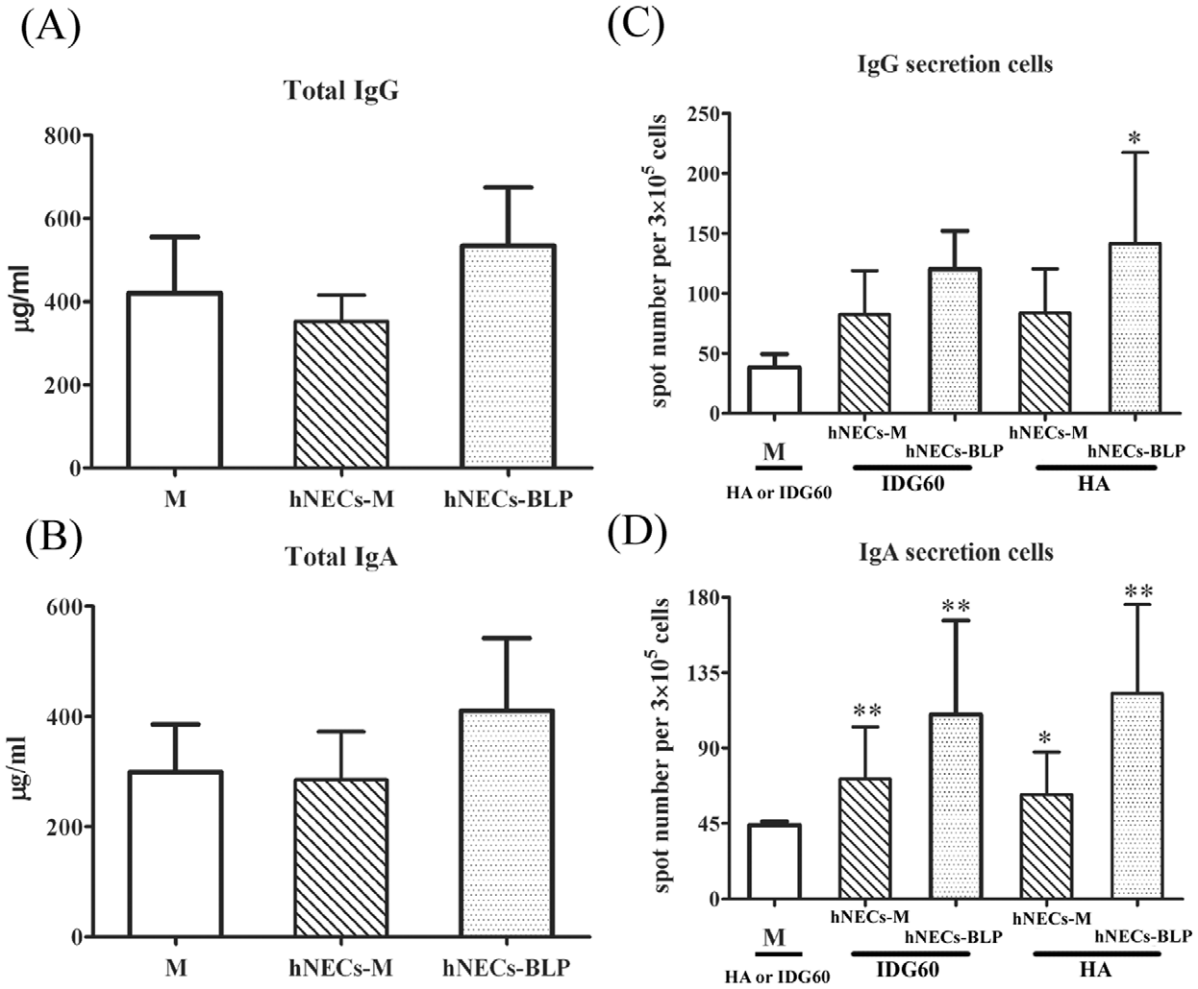
Antigen-specific immunoglobulin secretion cells increased by the CM from hNECs pretreated with BLPs. Leu-Leu-OMe-treated human PBLs were grown in the presence of CM from hNECs with (hNECs-BLP) or without BLP (hNECs-M) stimulation in the presence of soluble antigen IDG60 or HA for seven days. Total IgA (A) and total IgG (B) production in supernatants and antigen-specific IgA (C) or IgG (D) secreting cells were detected using ELISA and ELISpot, respectively. Representative ELISpot data were analyzed and represented as the mean spot number ± standard deviation (SD) per 3×10^5^ cells of antigen-specific secreted cells from three individuals performed in triplicate. Significant differences compared with medium-stimulated cells using *t*-tests are denoted by asterisks (*p<0.05 and **p<0.01).

Human PBLs from three healthy volunteers were incubated in the presence of conditioned medium from stimulated hNECs for seven days to determine whether hNECs secrete factors that are important in the total or antigen-specific antibody response. IDG60 and HA were used as the antigens in the *in vitro* immunization assay. Both conditioned and non-conditioned mediums exhibited a negligible effect on total IgG and IgA production (Fig. 4A and B). When IDG60 was added as the tested antigen, the number of IDG60-specific IgG secretion cells increased by 2.9-fold (*p* = 0.17) with the addition of non-conditioned medium and up to 3.5-fold (*p* = 0.21) in the presence of conditioned medium compared to PBLs cultured in RPMI medium alone (Fig. 4C). Consistently, the number of IDG60-specific IgA secretion cells was consistently and significantly increased by 1.6-fold (*p* = 0.009) and 2.6-fold (*p* = 0.0028), respectively (Fig. 4D). An analogous phenomenon was also observed for an HA-specific response. The number of HA-specific IgA or IgG secretion cells was significantly increased by the addition of conditioned medium (Fig. 4C and D). The number of HA-specific IgG and IgA secretion cells increased by 3.4- (*p* = 0.04) and 2.9- (*p* = 0.0031) folds, respectively.

### Mediators Responsible for hNECs Immuno-modulating Effect on Antigen Specific Response

**Figure 5:**
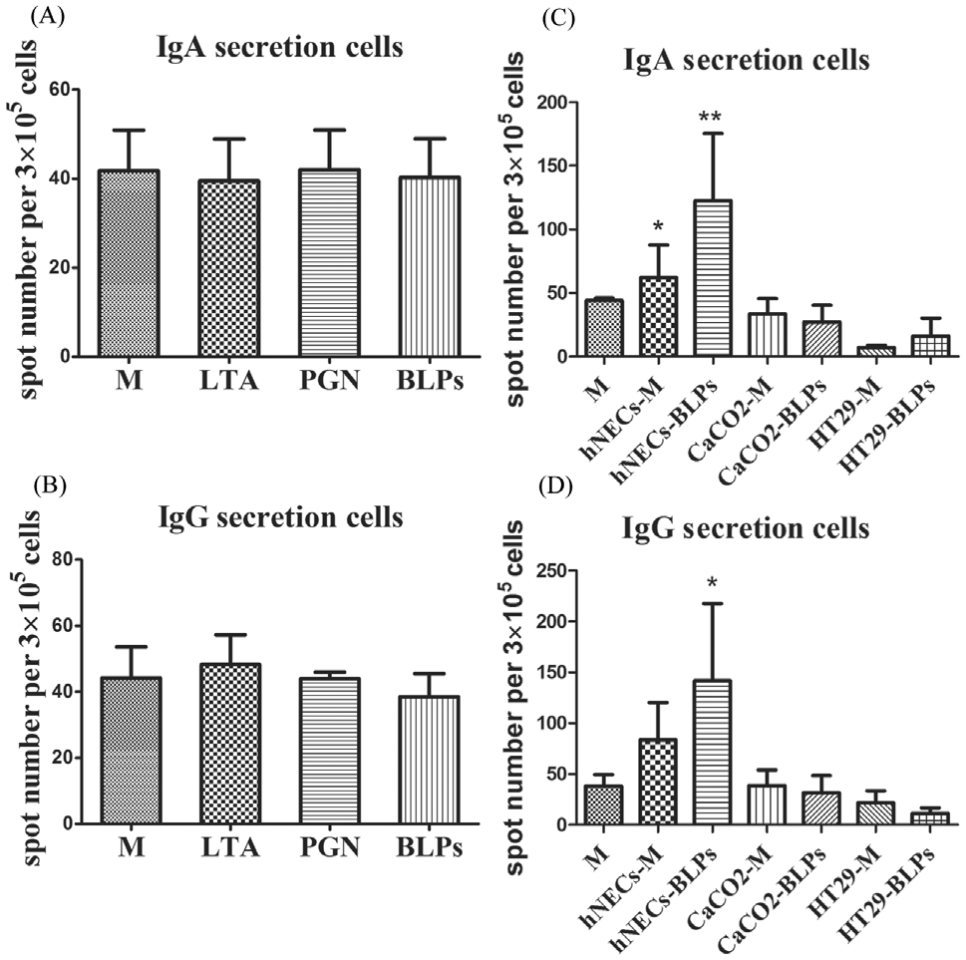
Antigen-specific response in TLR2 ligand stimulated PBLs and by the conditioned medium from BLPs-stimulated hNECs or intestinal cells. Human PBLs were cultured in the presence TLR2 ligands LTA, PGN or BLPs; or conditioned medium. Antigen-specific IgA (A and C) or IgG (B and D) secreting cells were detected by ELISpot. Significant differences compared with medium-stimulated cells using *t*-tests are denoted by asterisks (*p<0.05 and **p<0.01).

**Figure 6:**
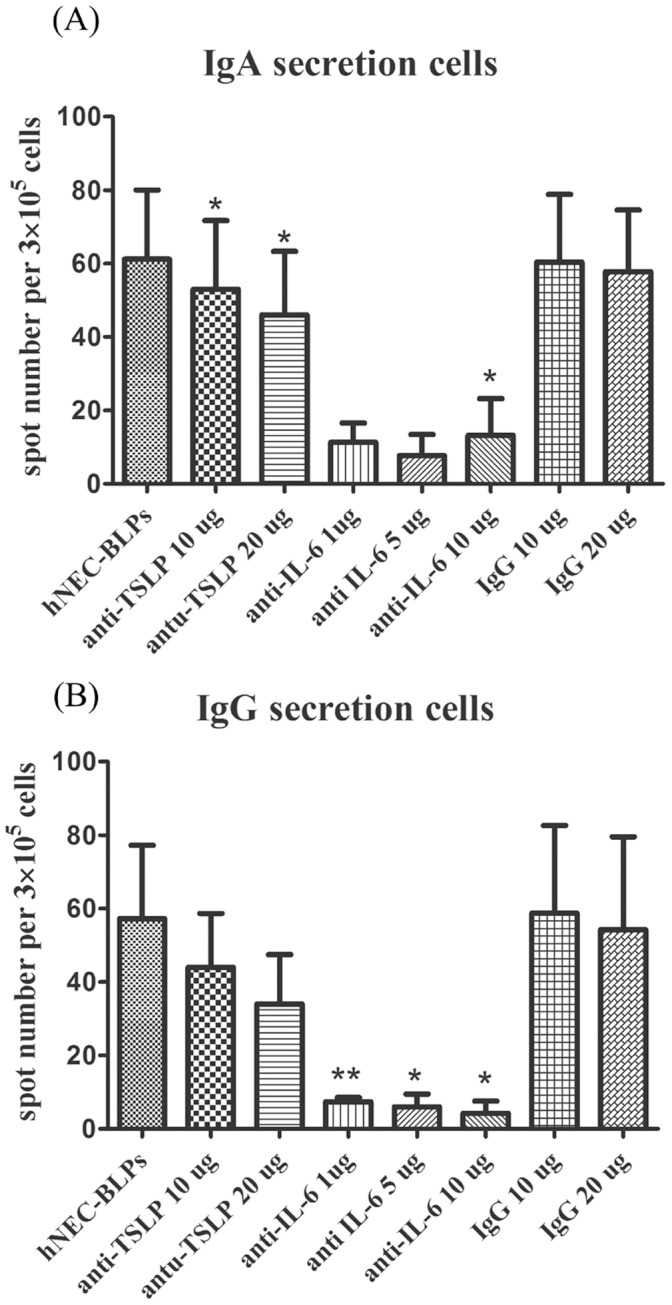
Antigen-specific immunoglobulin secretion cells analysis in the presence of anti-TSLP, anti-IL-6 and control IgG. Human PBLs were grown in the presence of conditioned medium from BLPs stimulated hNECs with or without neutralized antibodies. Antigen-specific IgA (A) or IgG (B) secreting cells were examined by ELISpot. Significant differences compared with medium-stimulated cells using *t*-tests are denoted by asterisks (*p<0.05 and **p<0.01).

To further examine if the observed enhancing effect on antibody production might also be exerted by epithelial cells from other anatomic locations and to test if the enhancing effect could be achieved following stimulation by other Toll-like receptor ligands, human intestinal epithelial cells lines HT29 and CaCO2 were tested in parallel experiments. The antibody-enhancing effects in *in vitro* immunization assay could not be observed by LTA, PGN or BLPs when adding directly in the absence of condition medium from hNEC (Fig. 5A and B). Most interestingly, antigen specific IgA or IgG production could be enhanced only by conditioned medium from BLPs stimulated hNECs, but not in BLPs stimulated human intestinal epithelial cells (Fig. 5C and D). In consistent with this finding was that the IL-6 or TSLP concentrations released form non-stimulated or BLPs stimulated human intestinal epithelial cells was less than the minimal detectable concentration 10 pg/ml, suggesting that these two cytokines, may be key mediators for stimulating antibody production. This hypothesis was supported by the finding that antigen specific IgA or IgG production can be blocked by the addition of IL-6 neutralizing antibody or inhibited by TSLP neutralizing antibody, but not control IgG in a dose-dependent manner (Figure 6A and B).

## Discussion

Nasopharynx-associated lymphoid tissue, so called Waldeyer’s ring in humans, plays an essential role in defensive immunity against microbial infections and is also a unique inductive site for B-cell responses and plasma cell generation [24]. Theoretically, intranasal immunization would be a non-invasive and efficient route in vaccine development [25]. Nevertheless, only limited information is available on the response of human nasal epithelial cells to the stimuli of microbial components and their effect on the adaptive immunity. We obtained hNECs from the sinus mucosa of patients with chronic rhinosinusitis used in *in vitro* epithelium cultures and experiments. Preliminary data indicated that hNECs constitutively produce various cytokines or chemokines, such as IL-8 or MCP-1, before stimulation. Although individual differences exist, six batches of cultures with comparable IL-8 and MCP-1 production levels were selected for subsequent analysis. Through the *in vitro* immunization assay, we identified IL-6 and TSLP as important mediators, while IL-6 plays a dominant role. IL-6 has been identified as one of the regulators for T cell proliferation, differentiation; and also Ig secretion by B cell [26]. In our experiments, the presence of IL-6 neutralizing antibodies may inhibit the T or B lymphocyte activation, resulting in the reduction of antigens specific IgA and IgG secreted cells detected by ELISpot. Moreover, the spot numbers presented in the presence of anti-IL-6 are less than unstimulated PBLs, suggesting that IL-6 may be the determinant in local immune reaction. Altogether, our results demonstrated that hNECs could exert an immuno-modulating effects on the specific IgA and IgG antibody response against bacterial or viral antigen and that IL-6 plus TSLP secreted from BLPs stimulated hNECs are key mediators in promoting antigen specific response.

A previous report demonstrated that BLPs can induce co-stimulatory molecule expression and cytokine production in human CD11c+ DCs through TLR2 activation [17]. Our results demonstrated that BLPs can also activate *in vitro* human DCs indirectly through interaction with epithelial cells. TSLP is a component released by stimulated hNECs and is an IL-7 family that stimulates DCs to become activated and subsequently skew T cells to Th2 polarization through the upregulation of IL-4, IL-13, and TNF-α in DCs [6]. Stimulation by IL-4, TNF-α, or TLR ligands can induce TSLP production in human airway and colonic epithelial cells [27], [28]. Although we did not measure IL-4 or TNF-α in our study, we did find that the TLR2 transcript in hNECs was upregulated, suggesting that TSLP expression may not be mediated through these cytokines but rather by direct TLR activation through BLPs. However, whether the modulating effect of BLPs on hNECs was achieved through TLR2 or in collaborating with other PAMPs in hNECs awaits further investigation. Moreover, TSLP may also enhance the barrier function of hNECs and increase the expression of tight junction proteins claudin-1,-4, and -7 as well as occludin [29]. These results suggest that TSLP plays a key role in the communication between hNECs and DCs. In our *in vitro* immunized PBLs experiment, addition of TSLP neutralizing antibodies achieved significant, but only partial inhibition on the antigen specific IgG or IgA production. It is possible that other Th2 polarizing cytokines such as IL-4 was present in the culturing condition [30]. To elucidate the degree of TSLP involvement in antigen specific response, further experiments such as eliminating IL-4 effects are needed.

*In vitro* immunization of PBLs has been adopted for the production of human monoclonal antibodies [31], [32] as well as the demonstration of antigen-specific IgM, IgG, and IgE [33]. Interestingly, we found that conditioned medium of BLPs-stimulated hNECs only enhanced the number of antigen-specific IgA or IgG producing cells, whereas the total immunoglobulin production was unaffected. One of the reason may be because BAFF and APRIL mRNA levels were constitutively high and not further upregulated by BLPs stimulation in cultured hNECs. On the other hand, BAFF secretion was increased in both BLPs stimulated hNECs and intestinal epithelial cells which were confirmed by dot blot (data not shown). This implied that BAFF secreted by epithelial cells alone are not enough to trigger antigen specific immune response. IL-6, IL-7, and IL-10 transcription was also upregulated in hNECs when co-cultured with BLPs-stimulated hNECs in a transwell apparatus, while the transcription of BAFF was upregulated in PBMCs (data not shown). These results indicate that B cell activating factors could also be derived from lymphocytes in addition to stimulated hNECs. Distinct from previous reports, we also found that an antigen-specific IgA response was enhanced by stimulated hNECs in addition to IgM, IgG, and IgE, suggesting that an epithelial-derived soluble mediator may assist with the IgA class switch signal. This information is helpful for the *in vitro* generation of antigen-specific human IgA antibodies.

BLPs were derived from acid and heat-killed non-recombinant *L. lactis*, which was formerly called Gram-positive enhancer matrix (GEM) [14]. BLPs contain bacterial peptidoglycan spheres that lack other intact cell wall components and intracellular materials, and could serve as a carrier for the delivery of protein antigens in mucosal immunizations [15], [16]. The BLPs were designed to non-covalently conjugate an antigen to the bacterial cell wall through fusion with the LysM-type peptidoglycan binding domains in the C-terminus of the lactococcal enzyme AcmA [14]. Interestingly, IDG60 of oral *S. mutans*, which lacks LysM, can also directly bind to BLPs through its native peptidoglycan binding domain. The BLP-IDG60 conjugate induced significantly stronger mucosal and systemic humoral immune responses in BALB/c mice after intranasal administration compared to soluble IDG60 alone. Similar results were obtained when HA was admixed with BLPs. Therefore, the binding of antigens may not always be necessary for the activating effects of BLPs on hNECs. These results agree with Saluja *et al*. [34]–[36], who demonstrated that HA admixed with BLPs enhances HA-specific immune responses in mice after intranasal, oral, and intramuscular delivery. BLPs are spheres of Gram positive bacteria and contain primarily peptidoglycan and lipoteichoic acids. Lipoteichoic acids being covalently linked to lipids within the cytoplasmic membrane are responsible for linking the peptidoglycan to the cytoplasmic membrane. Teichoic acids give the Gram positive spheres an overall negative charge due to the presence of phosphodiester bonds between teichoic acid monomers. Therefore, the direct binding of IDG60 to BLPs may involve the electrostatic linkage as opposed to a covalent conjugation. Such an electrostatic association between soluble protein antigens and TLR ligands has been shown to be important in enhancing immunogenicity [37], [38]. Steric arrangement of antigen and adjuvant is also important in antibody-generating response [38]. The results of the *in vitro* human PBL response indicated that stimulated hNECs can activate DCs to alter both Th1 and Th2 responses, which are characterized by the enhanced production of IFN-γ and IL-5, respectively. In agreement with the *in vitro* findings, both IgG2a and IgG1 were significantly elevated in mice following intranasal immunization of BLP-IDG60. Taken together, our results show that stimulation of hNECs by BLPs, results in the release of immuno-modulatory mediators, such as IL-6 and TSLP, which can activate mDCs to upregulate the expression of co-stimulatory molecules and enhance T cell proliferation and specific antibody production against bacterial or viral antigens. These results suggest that hNECs as the first line of mucosal lining against foreign invaders may exert active role in modulating both innate and adaptive immune responses in addition to acting as mucosal barriers.

In conclusion, the primary goal of the present study to test if hNECs may exert immuno-modulating effect on primary PBMCs to affect the production of antigen specific antibody responses has been verified. More than that, with properly designed antigen stimulation, hNECs may have more pluripotent effects in directing both innate and acquired immune response.

## Supporting Information

Figure S1 TLR2, TLR4, and GAPDH expression in human nasal and intestinal epithelial cells. Cells were harvested after 24 h with or without BLP stimulation. The transcription of TLR2 in hNECs (A) and the expression of TLR2, TLR4, and GAPDH in indicated epithelial cells (B) were examined by RT-PCR or western blot, respectively.
(TIF)

Table S1 Primer list.
(DOC)
