# Supplementary figures and images for: Activated Human Nasal Epithelial Cells Modulate Specific Antibody Response against Bacterial or Viral Antigens
Source: PLoS One. 2013 Feb 6;8(2):e55472. doi: 10.1371/journal.pone.0055472 (PMC3566203; doi:10.1371/journal.pone.0055472)

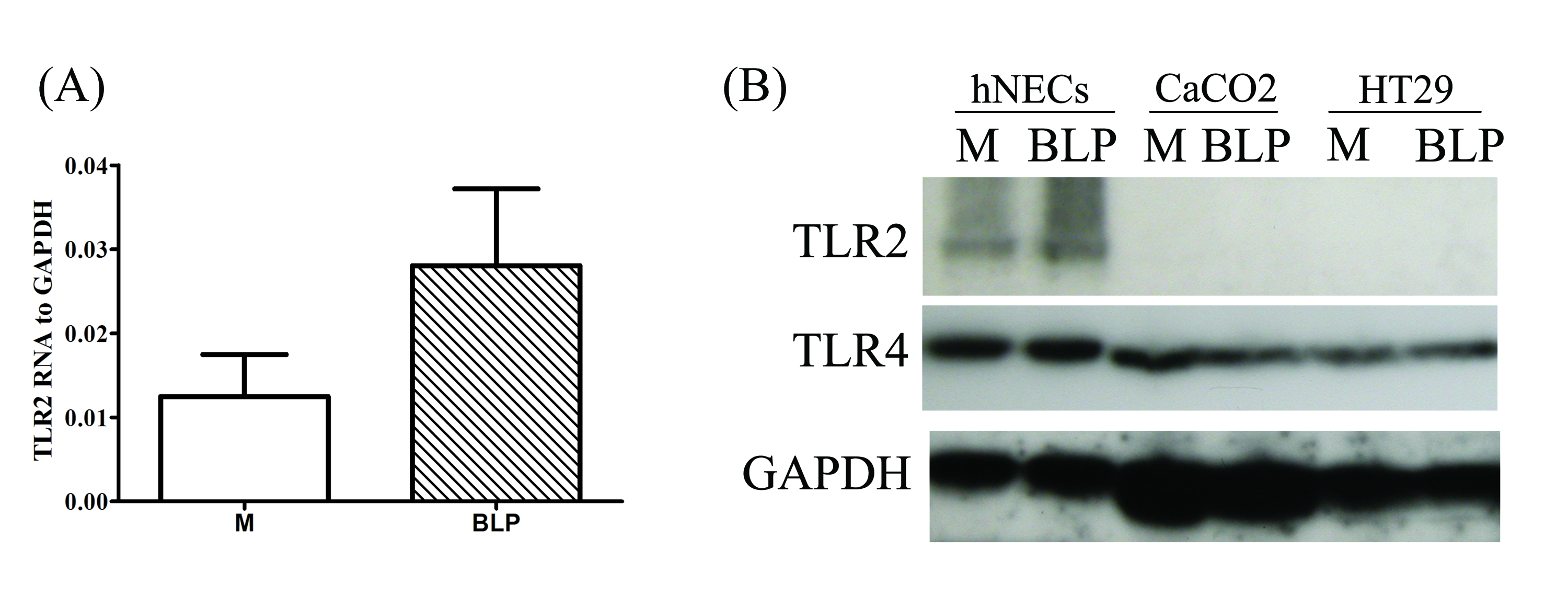

Supplement: Figure S1 — TLR2, TLR4, and GAPDH expression in human nasal and intestinal epithelial cells. Cells were harvested after 24 h with or without BLP stimulation. The transcription of TLR2 in hNECs (A) and the expression of TLR2, TLR4, and GAPDH in indicated epithelial cells (B) were examined by RT-PCR or western blot, respectively. (TIF) [file pone.0055472.s001.tif]
