# Supplementary material for: Activated Human Nasal Epithelial Cells Modulate Specific Antibody Response against Bacterial or Viral Antigens
Source: PLoS One. 2013 Feb 6;8(2):e55472. doi: 10.1371/journal.pone.0055472 (PMC3566203; doi:10.1371/journal.pone.0055472)
Supplement: Table S1 — Primer list. (DOC) [file pone.0055472.s002.doc]

**Table S1**: Primer list.

| GAPDH | F | 5′-GAAGGTGAAGGTCGGAGTC-3′ |
| --- | --- | --- |
|  | R | 5′-GAAGATGGTGATGGGATTTG-3′ |
| TSLP | F | 5′- TATGAGTGGGACCAAAAGTACCG-3′ |
|  | R | 5′- ACGCCACAATCCTTGTAATTGTG-3′ |
| CCL20 | F | 5′-GCGAATCAGAAGCAAGCAACT-3′ |
|  | R | 5′-GCATTGATGTCACAGCCTTCA-3′ |
| TLR2 | F | 5′-GTTAACAATCCGGAGGCTGC-3′ |
|  | R | 5′-TTGGGAATGCAGCCTGTTAC-3′ |
